# A randomized, double-blind, placebo-controlled study of rapid-acting intramuscular olanzapine in Japanese patients for schizophrenia with acute agitation

**DOI:** 10.1186/1471-244X-13-20

**Published:** 2013-01-11

**Authors:** Hideaki Katagiri, Shinji Fujikoshi, Takuya Suzuki, Kiyoshi Fujita, Naoya Sugiyama, Michihiro Takahashi, Juan-Carlos Gomez

**Affiliations:** 1 Lilly Research Laboratories Japan, Eli Lilly Japan K.K, Kobe, Japan; 2 Ayase Hospital, 6-3-1 Ayase, Adachi-ku, Tokyo, 120-0005, Japan; 3 Seishinkai Okehazama Hospital Fujita Kokoro Care Center, 3-879 Sakaecho Minamiyakata, Toyoake-shi, Aichi, 470-1168, Japan; 4 Numazu Chuo Hospital, 24-1 Nakasecho, Numazu-shi, Shizuoka, 410-0811, Japan; 5 Lilly Research Laboratories, Lilly Corporate Center, Indianapolis, IN, 46285, USA; 6 Takahashi Psychiatric Clinic, Ashiya, Hyogo, Japan

**Keywords:** Rapid-acting intramuscular olanzapine, Schizophrenia, Acute psychotic agitation, Japanese

## Abstract

**Background:**

Olanzapine rapid-acting intramuscular (IM) injection is an atypical antipsychotic drug already used overseas and recently approved in Japan. The objective of this study was to confirm the efficacy of rapid-acting IM olanzapine 10 mg was greater than IM placebo in patients with exacerbation of schizophrenia with acute psychotic agitation by comparing changes from baseline to 2 hours after the first IM injection, as measured by the Positive and Negative Syndrome Scale-Excited Component (PANSS-EC) total score.

**Methods:**

We conducted a placebo-controlled, randomized, double-blind, parallel-group study in Japanese patients diagnosed with schizophrenia according to the diagnostic criteria specified in the DSM-IV-TR. Patients were randomized to 2 treatment groups: IM olanzapine (10 mg) or IM placebo. The primary efficacy outcome was the change in PANSS-EC from baseline to 2 hours after the first IM injection. Treatment groups were compared with an analysis of variance model which included treatment and site as factors. During the 24-hour treatment period, safety was assessed by clinical examination and laboratory investigations, electrocardiograms, extrapyramidal symptoms scales, and recording spontaneously reported adverse events.

**Results:**

Of the 91 randomized patients, 90 patients (45 IM olanzapine-group; 45 IM placebo-group) were in the full analysis set. The mean change of PANSS-EC total score from baseline to 2 hours after the first IM injection (mean±standard deviation) was −9.2±4.5 for the IM olanzapine group and −2.8±5.6 for the IM placebo group. The difference between treatment groups was statistically significant (*p*<.001). There were no deaths, serious adverse events, treatment-emergent adverse events (TEAEs) leading to discontinuation, severe TEAEs, or instances of oversedation in this study. There were no statistically significant differences between treatment groups in the proportion of patients with potentially clinically significant changes in laboratory tests, vital signs (blood pressure and pulse rate), electrocardiograms, and treatment-emergent extrapyramidal symptoms.

**Conclusion:**

The efficacy of IM olanzapine 10 mg in patients with exacerbation of schizophrenia with acute psychotic agitation was greater than IM placebo in the primary efficacy measure, PANSS-EC. Intramuscular olanzapine 10 mg was shown to be generally safe and tolerable, and could be a new option for treatment of schizophrenia in Japan.

**Trial Registration:**

NCT00970281

## Background

Schizophrenia is a chronic disease, but in the acute phase agitation is common and may be accompanied by destructive and/or violent behavior [1-5]. In those cases, patients should be calmed as soon as possible to achieve a state of calm sufficient to minimize the risk posed to them or to others. Typical injections, such as a haloperidol injection, have been frequently used to calm patients with severe agitation [6]. However, intramuscular (IM) typical neuroleptics have been associated with undesirable side effects, including acute dystonia [7], malignant syndrome [8] and abnormal electrocardiograms including corrected QT interval (QTc) prolongation [9-11]. Currier and Simpson’s 2001 study suggested that treatment with risperidone liquid concentrate and oral lorazepam was a tolerable and comparable alternative to IM haloperidol and lorazepam for short-term treatment of agitated psychosis in patients who accept oral medications [12]. In addition, olanzapine orally disintegrating tablet versus risperidone oral solution yielded similar improvements in acutely agitated patients who accepted oral medication [13]. Oral medications are considered to be preferable to parenteral administration [14]. Therefore, oral solutions have become used more frequently than injections to calm patients who have agitation, but patients with severe agitation do not always take oral medications. Antipsychotic injections allow physicians to treat the patients, but also could be expected to calm patients more rapidly than oral medications [15]. Therefore, antipsychotic injections are still an option, especially to calm agitated patients who refuse to take medications.

A rapid-acting IM olanzapine was developed for rapid tranquilization of patients with acute-phase schizophrenia (exacerbation of schizophrenia with acute psychotic agitation) requiring parenteral drug therapy. Currently, IM olanzapine has been approved in more than 83 countries including in the United States and various countries in the European Union, and was approved in Japan in 2012. The original worldwide development of IM olanzapine did not include patients in Japan, so a Japan-specific development plan was undertaken. In Japan, oral olanzapine was approved in 2000 for the indication of schizophrenia, 2010 for improvement of manic symptoms in bipolar disorder, and in 2012 for improvement of depressive symptoms in bipolar disorder. Although introduction of oral atypical antipsychotics to Japan broadened treatment options for patients with schizophrenia, and rapid-acting injectable atypical antipsychotics only recently became available. Intramuscular olanzapine has a generally favorable profile with regard to extrapyramidal symptoms, QTc prolongation and oversedation [16-19]. In addition, IM olanzapine is positioned as one of the first-line agents for treatment of acute phase schizophrenia requiring IM pharmacotherapy out of Japan [4,20].

A randomized, placebo-controlled, double-blind study of IM olanzapine in Japanese patients with an exacerbation of schizophrenia with acute psychotic agitation was conducted. The primary objective of the study was to confirm the efficacy of IM olanzapine (10 mg) was greater than efficacy of IM placebo in Japanese patients who had exacerbation of schizophrenia with acute psychotic agitation. Efficacy was measured by comparing the change from baseline to 2 hours after the first IM injection in the Positive and Negative Syndrome Scale-Excited Component (PANSS-EC) total score [21,22]. We report the results of this study in this paper.

## Methods

### Study design and patient selection

This was a multicenter, placebo-controlled, randomized, double-blind, parallel-group study in Japanese patients diagnosed with schizophrenia according to the diagnostic criteria specified in the *Diagnostic and Statistical Manual of Mental Disorders, Fourth Edition, Text Revision* (DSM-IV-TR). Outpatients with an exacerbation of schizophrenia with acute psychotic agitation who required hospitalization at a regular doctor visit or in an emergency room, and inpatients with acute psychotic agitation were eligible for this study. Patients with acute psychotic agitation were defined as those who met any of following 3 criteria: patients whose agitation occurred or worsened within the prior 2 weeks, patients who were considered to require rapid tranquilization, or patients who needed careful consideration for examination or treatment (for example, more than 1 medical staff, special room).

The exclusion criteria in the current study were (1) patients whose agitation continued more than 2 weeks before providing informed consent, (2) patients whose agitation was caused by substance abuse, neurologic conditions or the comorbidity of mental retardation or personality disorders, and (3) patients who had inadequately controlled diabetes, or patients whose treatments for diabetes had been changed within 4 weeks before the first IM injection of the investigational product.

The study population was composed of patients who had met DSM-IV-TR criteria for schizophrenia, who were at least 20 years of age and less than 65 years of age who had exacerbation of schizophrenia with acute psychotic agitation, and with an Agitation-Calmness Evaluation Scale (ACES Eli Lilly and Company, Indianapolis, Indiana; ©1998, All rights reserved) score of 1 or 2 before the first IM injection of the investigational product. The ACES is a single-item scale developed by Eli Lilly and Company on which 1 indicates Marked Agitation; 2, Moderate Agitation; 3, Mild Agitation; 4, Normal; 5, Mild Calmness; 6, Moderate Calmness; 7, Marked Calmness; 8, Deep Sleep; and 9, Unarousable [16,23].

### Intervention

Use of drugs considered to affect the central nervous system was prohibited as a rule from 2 hours prior to the initial administration of the study drug to 24 hours after the administration. Conditional use of oral drugs was allowed. Oral administration of benzodiazepine hypnotics and anti-anxiety drugs was allowed only when the continued treatment with them was difficult to stop and a new treatment was required for adverse events excluding the period from 4 hours before to 2 hours after the administration. Anticholinergic agents were allowed from 2 hours before administration of the study drug to the end of the study only when the treatment was continuously used and could not be stopped or it was used as a new treatment for adverse events.

Patients were randomized to 2 treatment groups: IM olanzapine (10 mg) or IM placebo. All patients received at least 1 injection in the upper outer quadrant of the gluteus maximus muscles. If the investigator or subinvestigator(s) determined that the patient had no response or unsatisfactory efficacy after the first IM injection of the investigational product, the patient was permitted to receive a second IM injection. The second IM injection was administered at the same dose as the first IM injection, during the period from the completion of assessments scheduled for 2 hours after the first IM injection to 4 hours after the first IM injection.

### Efficacy and safety analysis

The primary efficacy measure was the change from baseline to 2 hours after the first IM injection as measured by PANSS-EC total score. The changes at 15, 30, 60, and 90 minutes and 24 hours after the administration were also compared between the treatment groups. PANSS-EC consists of the following 5 items: Excitement, Hostility, Tension, Uncooperativeness, and Poor Impulse Control.[21,22] Each item was rated on a scale from 1 (Absent) to 7 (Extreme). The sum of these ratings for all 5 items is defined as the PANSS-EC total score which ranged from 5 to 35. The secondary efficacy measures were ACES and the response to the investigational product. The proportion of patients who showed an ACES score of 4 (Normal) to 7 (Severe Sedation) at 30, 60, and 90 minutes and 2 and 24 hours after the initial administration was compared between treatment groups. The response to the investigational product was evaluated by the proportion of responders defined as patients with ≥40% decrease in the PANSS-EC total score at 2 hours after the first IM injection from baseline.

During the 24-hour treatment period, safety was assessed by clinical examination and laboratory investigations, and recording spontaneously reported adverse events. The severity of extrapyramidal symptoms was evaluated at baseline and 24 hours after the initial administration based on the Drug-Induced Extra-pyramidal Symptoms Scale (DIEPSS) [24]. The ECGs were performed during screening and at 2 and 24 hours after the first IM injection. The ACES was used to evaluate the safety of the investigational product in addition to its efficacy evaluation, a score of 8 (Deep Sleep) or 9 (Unarousable) was defined as oversedation.

### Statistical evaluation

A total of 90 patients (45 patients per group) was determined to be the necessary sample size to verify that the decrease in PANSS-EC was significantly greater in the IM olanzapine group than in the IM placebo group, by a Student’s *t*-test with a power of 90% at a 2-sided significance level of 5%. Considering the variation of the effect size in previous studies [25,26], total sample size was conservatively set at 90 patients (45 patients per group).

Efficacy and safety analyses were conducted on the full analysis set (FAS). This set principally included all data from all randomized patients receiving at least 1 dose of the study drug. The IM olanzapine group was compared with the IM placebo group using an analysis of variance (ANOVA) model which included treatment and site as factors. For proportions, a Fisher’s exact test was used. All statistical tests were performed at a 2-sided significance level of 5%. The approach to handling missing data was based on the last observation carried forward (LOCF).

The primary efficacy outcome was the LOCF change in PANSS-EC total score from baseline to 2 hours after the first IM injection. The LOCF change was compared between the IM olanzapine and IM placebo groups, using an ANOVA model which includes treatment and the site as factors. The LOCF change in the PANSS-EC total score from baseline to each evaluation timepoint within 2 hours after the first injection and to 24 hours after the first injection was compared between the 2 treatment groups.

A treatment-emergent adverse event (TEAE) was defined as an untoward medical occurrence that newly occurred or deteriorated after the first IM injection of the investigational product. Each occurrence was coded with reference to the *Medical Dictionary for Regulatory Activities (MedDRA)* (version 13.1). The proportion of patients with TEAEs, adverse drug reactions (ADRs) (defined as adverse events possibly related to the investigational product), serious adverse events (SAEs), TEAEs leading to discontinuation, and severe TEAEs were compared between the treatment groups. After the first IM injection, the proportion of patients with an ACES score of 8 (Deep Sleep) or 9 (Unarousable) were measured to assess oversedation. The proportions of patients with potentially clinically significant abnormal laboratory values, blood pressure, pulse rate, and ECGs were compared between the treatment groups. Treatment-emergent extrapyramidal symptoms were evaluated based on the DIEPSS score. Parkinsonism (Gait, Bradykinesia, Sialorrhea, Muscle Rigidity, and Tremor) was defined if a patient met 1 of the following 3 criteria (score ≥3 on 1 item, score ≥2 on 2 items, or increase of ≥3 from baseline). If a patient met 1 of the first 2 criteria at baseline, the patient was excluded from the analysis. Akathisia, Dystonia, and Dyskinesia were defined if a patient met 1 of 2 criteria (score ≥2 or increase of ≥2 from baseline). If a patient met the first criteria at baseline, the patient was excluded from the analysis.

### Ethics

The protocol of this study was prepared in accordance with the ethical principles from the Declaration of Helsinki and in compliance with the “Good Clinical Practice standards of pharmaceutical products” (GCP) and related laws and regulations. This study was conducted in compliance with the study protocol and GCP and related laws and regulations. Prior to the study conduct, the study protocol, case report forms, written explanation to patients and informed consent form as well as the conduct of the study were reviewed and approved by the institutional review boards of the respective study sites. The name of the investigational review boards can be found in the Ethics approval section at the end of this manuscript. Prior to initiation of the procedure and study drug administration, the investigators or subinvestigators obtained consent to participate in the study from the patients or their legal representatives by their free wills. When the study was conducted by the consent of a legal representative, the investigator or subinvestigator tried to obtain an additional consent from the patient once he/she became able to give consent. If the patient refused to give additional consent or he/she requested withdrawal from the study, he/she was able to be immediately withdrawn from the study.

## Results

Of the 91 randomized patients in this study, 90 patients (45 IM olanzapine-group patients; 45 IM placebo-group patients) were in the FAS. One patient in the randomized group was excluded from the FAS analysis due to discontinuation by physician’s decision before the first IM injection. Eighty-nine patients (45 IM olanzapine-group patients; 44 IM placebo-group patients) were used for the primary efficacy analysis. One patient in the FAS group was excluded from the efficacy analysis because of a problem in the maintenance of the blind. To be statistically conservative, the FAS used for the safety analysis included all patients who received study drug.

Table 1 summarizes the demographic characteristics for the FAS by treatment group and in total. The mean age was 46.7 years (range, 20–65 years); mean body mass index, 23.4 kg/m^2^ (range, 14.3–38.4 kg/m^2^); and mean age at onset, 25.9 years (range, 15–57 years). The proportion of male patients was 46.7% in the IM olanzapine group and 51.1% in the IM placebo group. Six of 90 patients (6.7%) had diabetes mellitus at baseline (IM olanzapine, historical n=1, pre-existing n=2; IM placebo, historical n=0, pre-existing n=3). No statistically significant differences were seen between the IM olanzapine and IM placebo groups at baseline in any demographic category. The baseline PANSS-EC total score (mean ± standard deviation [SD]) was 23.5±6.1 in the IM olanzapine group and 23.3±4.9 in the IM placebo group (*p*=.638) (Table 2). The baseline ACES score (mean ± SD) was 1.6±0.5 in the IM olanzapine group and 1.6±0.5 in the IM placebo group (*p*=.741). The baseline values showed no significant difference between the 2 treatment groups.

**Table 1:** Patient demographics

	**Item**	**IM Olz, 10 mg**	**IM Placebo**	**Total**	***p *value**^**a**^
		**(N=45)**	**(N=45)**	**(N=90)**	
Age, years	N	45	45	90	.812
	Mean	46.4	47.0	46.7	
	SD	11.7	12.1	11.9	
	Min	20	23	20	
	Median	50.0	45.0	47.0	
	Max	63	65	65	
Gender, n (%)	Male	21 (46.7)	23 (51.1)	44 (48.9)	.833
	Female	24 (53.3)	22 (48.9)	46 (51.1)	
BMI, kg/m^2^	N	45	45	90	.360
	Mean	23.9	23.0	23.4	
	SD	4.9	4.1	4.5	
	Min	16.1	14.3	14.3	
	Median	23.4	22.5	23.0	
	Max	38.4	35.1	38.4	
Age at onset, years	N	44	45	89	.215
	Mean	24.7	27.2	25.9	
	SD	9.4	9.3	9.4	
	Min	15	16	15	
	Median	22.0	25.0	23.0	
	Max	49	57	57	

Abbreviations: BMI=body mass index; IM Olz=intramuscular olanzapine; Max=maximum; Min=minimum; N= number of patients in analysis set; n= number of patients in subset; SD=standard deviation.

^a^*p* value: Student's *t* test for continuous data, Fisher's exact test for categorical data.

**Table 2:** Primary and secondary analyses, change from baseline to 2-hour timepoint (LOCF)

	**IM Olz, 10 mg (N=45)**			**IM Placebo (N=44)**			***p *value**^**b**^
	**Mean (points)**	**SD**	**95%CI**	**Mean (points)**	**SD**	**95%CI**	
Baseline							
PANSS-EC total	23.5	6.1	21.7, 25.4	23.3	4.9	21.8, 24.8	.638
Poor impulse control	4.8	1.2	4.5, 5.2	4.7	1.2	4.3, 5.0	.336
Tension	4.7	1.6	4.2, 5.2	4.7	1.1	4.3, 5.0	.722
Hostility	4.6	1.6	4.1, 5.1	4.6	1.3	4.2, 5.0	.912
Uncooperativeness	4.7	1.3	4.3, 5.1	4.8	1.2	4.4, 5.1	.791
Excitement	4.7	1.2	4.4, 5.1	4.6	1.0	4.3, 4.9	.385
ACES^a^	1.6	0.5	1.5, 1.7	1.6	0.5	1.5,1.8	.741
2 hours							
PANSS-EC total	14.3	6.0	12.5, 16.1	20.5	7.5	18.3, 22.8	
Poor impulse control	3.0	1.3	2.6, 3.4	4.1	1.5	3.7, 4.6	
Tension	2.9	1.2	2.5, 3.2	4.1	1.7	3.6, 4.6	
Hostility	2.8	1.3	2.4, 3.1	4.0	1.7	3.5, 4.5	
Uncooperativeness	2.9	1.4	2.5, 3.3	4.3	1.6	3.8, 4.8	
Excitement	2.8	1.2	2.4, 3.2	4.0	1.5	3.5, 4.4	
ACES	3.5	1.7	2.9, 4.0	2.2	1.3	1.8, 2.6	
Change, baseline-2 hours							
PANSS-EC Total	−9.2	4.5	−10.6, -7.9	−2.8	5.6	−4.5, -1.1	<.001
Poor impulse control	−1.9	1.0	−2.2, -1.6	−0.6	1.2	−0.9, -0.2	<.001
Tension	−1.8	1.1	−2.2, -1.5	−0.6	1.1	−0.9, -0.2	<.001
Hostility	−1.8	1.2	−2.2, -1.5	−0.6	1.5	−1.0, -0.1	<.001
Uncooperativeness	−1.7	0.9	−2.0, -1.5	−0.5	1.2	−0.8, -0.1	<.001
Excitement	−1.9	1.1	−2.3, -1.6	−0.6	1.4	−1.0, -0.2	<.001
ACES	1.9	1.5	1.4, 2.3	0.6	1.1	0.2, 0.9	<.001

Abbreviations: ACES=Agitation-Calmness Evaluation Scale; CI=confidence interval; IM Olz=intramuscular olanzapine; LOCF=last observation carried forward [analysis]; N=number of patients with a baseline and a value at the time (LOCF); PANSS-EC=Positive and Negative Syndrome Scale-Excited Component.

^a^At baseline placebo n=45.

^b^*p* value: ANOVA model which includes Treatment and Site as factors.

The primary efficacy endpoint, the mean change of PANSS-EC total score from baseline to 2 hours after the first IM injection was −9.2±4.5 for the IM olanzapine group and -2.8±5.6 for the IM placebo group (Table 2). The difference between treatment groups was statistically significant (*p*<.001) for the mean change of PANSS-EC total score from baseline to 2 hours. At the 2-hour timepoint, the IM olanzapine group showed a significant decrease in all PANSS-EC individual item scores compared with the IM placebo group.

**Figure 1:**
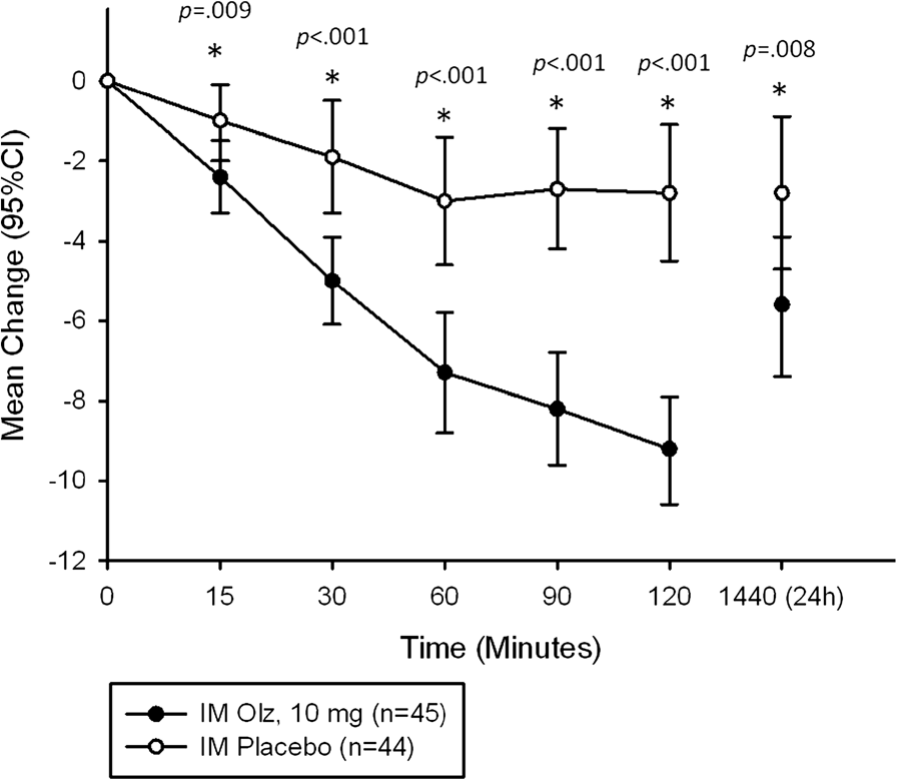
**Time-course change from baseline in PANSS-EC total score up to 24 hours after administration.**PANSS-EC total score for timepoint-wise change from baseline to 24 hours after the first IM injection, full analysis set, IM Olz, 10 mg (n=45), IM Placebo (n=44). Abbreviations: CI=confidence interval; IM Olz=intramuscular olanzapine *statistically significant difference between groups.

The change from baseline to each evaluation timepoint (15, 30, 60 and 90 minutes, and 2 and 24 hours after the first IM injection) in PANSS-EC total scores was a secondary efficacy endpoint. At every timepoint, statistically significant differences were observed between IM olanzapine and IM placebo groups (*p*<.001) (Figure 1). The maximum change in PANSS-EC total score in the IM olanzapine group was observed at the 2-hour timepoint. At the 24-hour timepoint the mean change in PANSS-EC total score in the IM olanzapine group decreased to −5.6 (from −9.2 at 2 hours), while IM placebo group remained at -2.8 (from −2.8 at 2 hours) (*p*=.008).

At 2 hours after the first IM injection, the proportion of responders (≥40% decrease in the PANSS-EC total score) was 40% (18/45 patients) in the IM olanzapine group and 13.6% (6/44 patients) in the IM placebo group. The difference between treatment groups in the proportion of responders was statistically significant (*p*=.008).

At 2 hours after the first IM injection the mean ACES score for IM olanzapine group was 3.5±1.7 (n=45) and in the IM placebo group the mean was 2.2±1.3 (n=44) (Table 2). The proportion of patients with the ACES score of 4 (Normal) to 7 (Marked Calmness) was evaluated at 2 hours after the first IM injection. ACES scores fell between 4 and 7 in 40% (18/45) of the IM olanzapine group and 13.6% (6/44) of the IM placebo group (*p*=.008). The mean change from baseline to 2 hours in ACES score for IM olanzapine (1.9±1.5) and IM placebo (0.6±1.1) groups was statistically significantly different between groups (*p*<.001).

Twenty patients (44.4%) in the IM olanzapine group received a second IM olanzapine injection (10 mg) after the first IM injection. Twenty-six patients (57.8%) in the IM placebo group received the second IM injection after the first IM injection (*p*=.292).

Ninety patients (45 IM olanzapine-group patients; 45 IM placebo-group patients) were analyzed for the safety measures. Table 3 contains an overview of adverse events reported during the study. Treatment-emergent adverse events were reported in 19 of the 90 patients during the study. Thirteen patients (28.9%) were in the IM olanzapine group, and 6 patients (13.3%) were in the IM placebo group. Only 2 TEAEs were reported in more than 1 patient, somnolence (IM olanzapine, n=7 [15.6%]; IM placebo, n=2 [4.4%]; *p*=.157) and blood urine present (IM olanzapine, n=0; IM placebo, n=2 [4.4%]; *p*=.494). There were no significant differences in proportion of patients with TEAEs between treatment groups. Severity of all TEAEs was mild, except orthostatic hypotension (IM olanzapine, n=1) and blood creatine phosphokinase increased (IM placebo, n=1), which were moderate. There were no statistically significant differences between the IM olanzapine and IM placebo groups in the proportion of patients with TEAEs (*p*=.120) or ADRs (*p*=.230). There were no deaths, SAEs, TEAEs leading to discontinuation, severe TEAEs, or instances of oversedation in this study.

**Table 3:** Adverse events

	**IM Olz, 10 mg (N=45)**	**IM Placebo (N=45)**	
**Item**	**n (%)**	**n (%)**	***p *value**^**a**^
TEAE	13 (28.9)	6 (13.3)	.120
ADR	9 (20.0)	4 (8.9)	.230
TEAE leading to discontinuation	0 (0.0)	0 (0.0)	———
Severe TEAE	0 (0.0)	0 (0.0)	———
Serious adverse event	0 (0.0)	0 (0.0)	———

Abbreviations: ADR= adverse drug reaction; IM Olz=intramuscular olanzapine; N= number of patients in the full analysis set; n=number of patients with ≥1 event;TEAE=treatment-emergent adverse event.

^a^*p* value: Fisher's exact test.

Treatment-emergent extrapyramidal symptoms such as Parkinsonism, Akathisia, Dystonia, and Dyskinesia were evaluated based on DIEPSS. Parkinsonism was observed in the IM olanzapine group (2/43 patients, 4.7%), and in the IM placebo group (3/44 patients, 6.8%) (*p*=1.000). There were no incidences of treatment-emergent extrapyramidal symptoms of Akathisia, Dystonia, or Dyskinesia at 24 hours after the first IM injections. There were no significant differences between the IM olanzapine and IM placebo groups in the proportion of patients using benzodiazepines or anticholinergics.

There were no statistically significant differences between treatment groups in the proportion of patients with potentially clinically significant changes in laboratory tests, vital signs (blood pressure and pulse rate), and ECGs. Two IM olanzapine group patients had a QT interval corrected for heart rate using Fridericia’s formula (QTcF) increase >30 msec at 2 hours and 1 IM olanzapine patient had an increase >30 msec at 24 hours after the IM injection. No patients in the IM placebo group showed a QTcF increase >30 msec at 2 or 24 hours after the IM injection. No patient, at any timepoint, showed an increase greater than 60 msec, or a QTcF greater than 480 msec. The mean change from baseline of QTcF was 1.6 msec for 2 hours, -2.6 msec for 24 hours for the IM olanzapine group. The mean change from baseline of QTcF was 4.1 msec for 2 hours, and −1.1 msec for 24 hours for the IM placebo group. These changes were not clinically significant and there were no statistically significant differences between the groups.

## Discussion

This was a placebo-controlled, double-blind confirmatory study of rapid-acting IM olanzapine in acutely agitated patients with schizophrenia. The primary objective of the study was to confirm the efficacy of IM olanzapine (10 mg) was greater than IM placebo in Japanese patients who had exacerbation of schizophrenia with acute psychotic agitation by comparing changes from baseline to 2 hours after the first IM injection, as measured by PANSS-EC total score. The study results showed a statistically significant difference between the IM olanzapine and IM placebo groups in the change from baseline to 2 hours in PANSS-EC total score (*p*<.001).

Onset of action was fast, the baseline to each evaluation timepoint in PANSS-EC total scores was significantly different between the IM olanzapine and IM placebo groups starting at 15 minutes. The efficacy of IM olanzapine has been demonstrated previously in overseas clinical studies [16,17], and now in the current study in Japanese patients.

This analysis did not show a statistically significant difference in the incidence of TEAE between the IM olanzapine group and the IM placebo group, and none of the events occurred at a significantly higher incidence in the IM olanzapine group compared with the IM placebo group. Somnolence was reported more often in IM olanzapine group patients but this difference between groups was not statistically significant. This may have been due to the sample sizes. Clinically relevant change was not observed with clinical laboratory data, vital signs, and ECG in patients treated with IM olanzapine. There were no patients who experienced oversedation as measured by ACES. Treatment-emergent extrapyramidal symptoms did occur during this study; however, the proportion in the placebo group was higher (4.7% olanzapine versus 6.8% placebo, *p*=1.000). Metabolic adverse events associated with glucose metabolism are often discussed as potential risks of olanzapine treatment [27]. Although there were no clear signals of adverse events or laboratory changes associated with glucose metabolism in this study, it is important to monitor patients for adverse events and laboratory changes associated with glucose metabolism in clinical practice.

There were several limitations to this study. The current study’s sample size was the necessary size to verify that the decrease in PANSS-EC was significant; however a larger sample size would have provided a better representation of the real-world patient population. The patients enrolled in this study were inpatients or outpatients who required hospitalization, and there were very few treatment naïve patients. Patients were required to show a minimum level of cooperativeness with study procedures, so the sample may not represent very severely agitated or combative patients with schizophrenia.

The previous confirmative study in Japan did not have a successful efficacy result [25]. One of the potential reasons for the lack of a statistical difference in the primary endpoint of the previous study was the inclusion of patients unlikely to show a drug-placebo separation, like patients with chronic agitation who are resistant to treatment or patients with nonpsychotic agitation who may respond to environmental interventions. In the current study as opposed to the previous study, additional inclusion and exclusion criteria were added. The exacerbation of schizophrenia with acute psychotic agitation was added as an inclusion criterion, and continuous agitation in patients for at least 2 weeks duration before informed consent was to be signed was added as an exclusion criterion. This was done to exclude patients who were resistant to treatment and showed high PANSS-EC total score chronically. In addition, to exclude patients with nonpsychotic agitation who could respond to environmental interventions, we set some specific exclusion criteria, such as the comorbidity of mental retardation or personality disorders that are more likely to show nonpsychotic reactive excitement and placebo response. These changes in inclusion and exclusion criteria have likely contributed to the current successful results.

## Conclusion

It was confirmed that the efficacy of IM olanzapine 10 mg was greater than IM placebo in patients who had exacerbation of schizophrenia with acute psychotic agitation as evaluated by the primary efficacy measure, PANSS-EC. There were no deaths, SAEs, TEAEs leading to discontinuation, severe TEAEs, or instances of oversedation in this study. Intramuscular olanzapine 10 mg was shown to be generally safe and tolerable in acutely agitated patients with schizophrenia.

Olanzapine rapid IM injection preparation demonstrated efficacy and safety in patients who had exacerbation of schizophrenia with acute psychotic agitation, and it could be a new option for treatment of schizophrenia with severe agitation, which cannot be treated with oral medication, in Japan.

### Ethics approvals

The following institutional review boards provided ethical approval for the study: Sapporo Skin Clinic Institutional Review Board, Institutional Review Board of Iwata Hospital, Tokushukai Group Institutional Review Board, Institutional Review Board of Kimura Hospital, Aichi Medical Association Institutional Review Board, Institutional Review Board of Hannan Hospital, Nara Medical University Hospital Institutional Review Board, Joint Institutional Review Board of Asai Hospital & Shiratori Hospital, Institutional Review Board of Yuge Hospital, Himorogi Psychiatric Institute Institutional Review Board, Institutional Review Board of Arakaki Hospital, Institutional Review Board of Negishi Hospital, and Institutional Review Board of Nishigahara Hospital.

## Competing interests

Dr. Katagiri is a full-time employee of Eli Lilly Japan K.K. and stockholder of Eli Lilly and Company. Mr. Fujikoshi is a full-time employee of Eli Lilly Japan K.K. and stockholder of Eli Lilly and Company. Dr. Takahashi is a part-time employee of Eli Lilly Japan K.K. and stockholder of Eli Lilly and Company. Dr. Gomez is a full-time employee of Eli Lilly and a stockholder of Eli Lilly and Company. Dr. Suzuki has received honorarium for lecturing/speaking from Eli Lilly Japan K.K., Dainippon Sumitomo Pharma, and Meiji Seika Pharma within the past 5 years. Dr. Fujita has received speaker’s honorarium from Eli Lilly Japan K.K., Otsuka Pharmaceutical, Janssen Pharma, Dainippon Sumitomo Pharma, GlaxoSmithKline, Pfizer, Mochida Pharmaceutical, Novartis, and Astellas Pharma within the past 5 years. Dr. Sugiyama has received speaker’s honorarium from and serves as a consultant to Eli Lilly Japan K.K.

## Authors’ contributions

HK participated in the conception and design of the study, analyzed and interpreted the data and drafted the manuscript. MT and SF participated in the conception of the study and analyzed and interpreted the data. JCG participated in the conception and design of the study, interpretation of the data and drafting of the manuscript. TS and KF were investigators in the study and participated in the acquisition of data. NS participated in the conception and design of the study. All authors participated in writing and editing the manuscript. All authors read and approved the final manuscript.

## Pre-publication history

The pre-publication history for this paper can be accessed here:

http://www.biomedcentral.com/1471-244X/13/20/prepub
