# Erratum: A randomized, double-blind, placebo-controlled study of rapid-acting intramuscular olanzapine in Japanese patients for schizophrenia with acute agitation

**DOI:** 10.1186/s12888-014-0313-9

**Published:** 2014-12-10

**Authors:** Hideaki Katagiri, Shinji Fujikoshi, Takuya Suzuki, Kiyoshi Fujita, Naoya Sugiyama, Michihiro Takahashi, Juan-Carlos Gomez

**Affiliations:** Lilly Research Laboratories Japan, Eli Lilly Japan K.K, Kobe, Japan; Ayase Hospital, 6-3-1 Ayase, Adachi-ku, Tokyo, 120-0005 Japan; Seishinkai Okehazama Hospital Fujita Kokoro Care Center, 3-879 Sakaecho Minamiyakata, Toyoake-shi, Aichi 470-1168 Japan; Numazu Chuo Hospital, 24-1 Nakasecho, Numazu-shi, Shizuoka 410-0811 Japan; Lilly Research Laboratories, Lilly Corporate Center, Indianapolis, IN 46285 USA; Takahashi Psychiatric Clinic, Ashiya, Hyogo Japan

## Correction

After the publication of this work [1], we became aware that it contains inaccurate description. The correct description on page 5 was “At every timepoint, statistically significant differences were observed between IM olanzapine and IM placebo groups (*p* < .01) (Figure one)”, and not “At every timepoint, statistically significant differences were observed between IM olanzapine and IM placebo groups (*p* < .001) (Figure one).” We regret any inconvenience that this inaccuracy might have caused.
